# Supplementary material for: Crystallization of molecular layers produced under confinement onto a surface
Source: Nat Commun. 2024 Mar 5;15:2015. doi: 10.1038/s41467-024-45900-0 (PMC10914826; doi:10.1038/s41467-024-45900-0)
Supplement: Supplementary file 1 — Supplementary Information [file 41467_2024_45900_MOESM1_ESM.pdf]

Supplementary Information

Crystallization of Molecular Layers produced Under Confinement onto a surface

Jincheng Tong[1*], Nathan de Bruyn[1], Adriana Alieva[1], Elizabeth. J. Legge[2,3], Matthew Boyes[1], Xiuju Song[1], Alvin J. Walisinghe[4], Andrew J. Pollard[2], Michael W. Anderson[1,4], Thomas Vetter[5], Manuel Melle-Franco[6], Cinzia Casiraghi[1*]

[1]Department of Chemistry, University of Manchester; Manchester, M13 9PL, UK.

[2]National Physical Laboratory; Teddington, Middlesex, TW11 0LW, UK.

[3]Advanced Technology Institute, University of Surrey; Guildford, Surrey, GU2 7XH, UK.

[4]Curtin Institute for Computation, School for Molecular and Life Sciences, Curtin University, Perth Western Australia 6845, Australia

[5]Department of Chemical Engineering and Analytical Sciences, University of Manchester; Manchester, M1 3AL, UK.

[6]CICECO—Aveiro Institute of Materials, Department of Chemistry, University of Aveiro; Aveiro, 3810-193, Portugal.

*Emails: tongjincheng@outlook.com (J.T.); cinzia.casiraghi@manchester.ac.uk (C.C.)

- **Supplementary Figures and Discussions**

Supplementary Section 1. Drop casting vs gas blowing coating

Supplementary Section 2. Characterization of glycine crystals obtained by gas blowing coating

Supplementary Section 2.1 Glycine crystals deposited at different pressures

Supplementary Section 2.2 Glycine crystals deposited by using different concentrations at 1.5 bar

Supplementary Section 2.3 Stability of glycine nanocrystals

Supplementary Section 3. Computer modelling of glycine

Supplementary Section 3.1 Intermolecular energies of glycine polymorphs calculation

Supplementary Section 3.2 Morphology mapping by CrystalGrower

Supplementary Section 4. Gas blowing coating of other molecules

Supplementary Section 4.1 Benzamide

Supplementary Section 4.2 DL-methionine

Supplementary Section 4.3 D-mannitol

Supplementary Section 4.4 MOF-5

- **Supplementary References**

Supplementary Figures and Discussions

Supplementary Section 1. Drop casting vs gas blowing coating

In this section we show that the gas blowing coating produces very different crystals, compared to those obtained by simple drop casting, as previously observed for organic semiconductors.[1]

Drop casting of 0.5 M glycine on the Ar plasma treated SiO_2/Si substrate results in dendritic crystals, **Supplementary Fig. 1**. Raman spectroscopy shows that both α- and β-glycine forms are formed, hence drop casting does not allow any control on the size, shape and crystal structure. Crystallization by gas blowing coating was repeated exactly under the same experimental conditions, i.e. 0.5 M glycine solution was deposited on an Ar plasma treated SiO_2/Si substrate, at different pressures (0.1-2 bar). At a pressure of 0.1 bar, an elongated wet film was produced uniformly on the substrate. Crystallization started from the edge of the elongated wet film, and then the crystals grew rapidly to the middle, in the direction perpendicular to the gas-flow direction (**Supplementary Fig. 2a**).

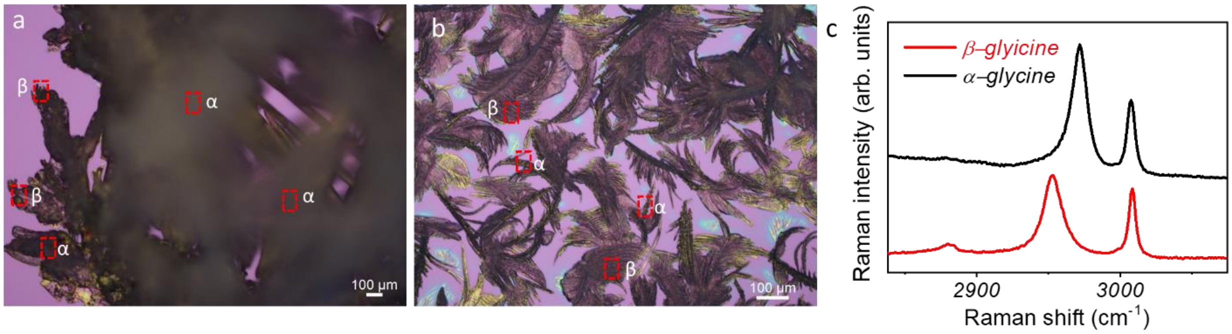

Supplementary Fig. 1: Optical image of glycine crystals obtained by drop casting of 0.5 M glycine aqueous solution on: (a) bare SiO_2/Si substrate and (b) Ar plasma treated SiO_2/Si substrate.

(c) Representative Raman spectra of the glycine polymorphs made by drop casting, measured on the crystals indicated in panels a and b.

By increasing the pressure to 0.2 bar, the lateral size of the leave-like crystals gets reduced (**Supplementary Fig. 2b**). The thickness of the crystal, measured by AFM, is above 1 µm, while the width is around 5 µm (**Supplementary Fig. 2c**). The corresponding Raman map shows that the single crystal is β-glycine (**Supplementary Fig. 2d**). At a pressure of 0.3 bar, the leave-like crystals morphology disappears: spherulitic crystals with thickness above 250 nm and with diameter approaching ~500 µm are observed (**Supplementary Fig. 3 a-b**). The center of the spherulitic crystal is compact and it is likely the point where nucleation starts (**Supplementary Fig. 3c**). By further increasing the pressure to 0.5 bar, the density of the spherulites increases, while the thickness decreases to ~200 nm. The diameter is around 250 µm (**Supplementary Fig. 3 d-e**). Raman map of the peak at 2953 cm^{-1} indicates that the obtained spherulites are β-glycine (**Supplementary Fig. 3f**). Increasing the pressure to 0.7 bar, the spherulites boundaries appear not well-defined as compared to the crystals obtained at 0.5 bar (**Supplementary Fig. 3g**). The crystals branches are now made of individual nanoparticles following defined directions (**Supplementary Fig. 3i**). At the pressure of 1 bar, a film of randomly distributed nanoparticles is obtained (**Supplementary Fig. 3 j-k**), indicating that the thickness of the wet film was somehow disrupted because of the high pressure used. This should give a higher nucleation density - however, as the nanoparticles are still linked together, they are not grown yet from individual puddles. Despite the strong change in morphology, Raman mapping shows the crystals to be still β-glycine (**Supplementary Fig. 3l**). At the pressure of 2 bar (**Supplementary Fig. 3 m-n**), isolated

nanoparticles of single crystals with clear facets are obtained (**Supplementary Fig. 3o**). The crystals are largely isolated, indicating that they were crystallized from individual puddles.

Drop casting of 0.01 M glycine on an Ar plasma treated SiO_2/Si substrate gives similar results obtained with the 0.5 M solution (Supplementary Fig. 1). **Supplementary Fig. 4** shows the AFM image of the sample, confirming that gas blowing coating allows to overcome dendritic (i.e. uncontrolled) growth, and to obtain individual crystals with defined size and shape (main text).

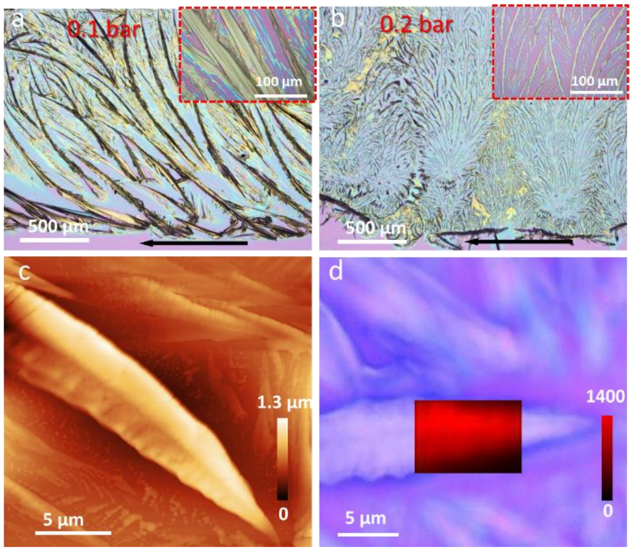

Supplementary Fig. 2. Optical images of crystals obtained by gas blowing coating at a pressure of: (a) 0.1 and (b) 0.2 bar. The insets show higher magnification images of the well-aligned crystals. The black arrow indicates the gas blowing coating direction. (c) AFM image and (d) Raman map of the intensity of the peak at 2953 cm^{-1}, fingerprint of the β-glycine form.

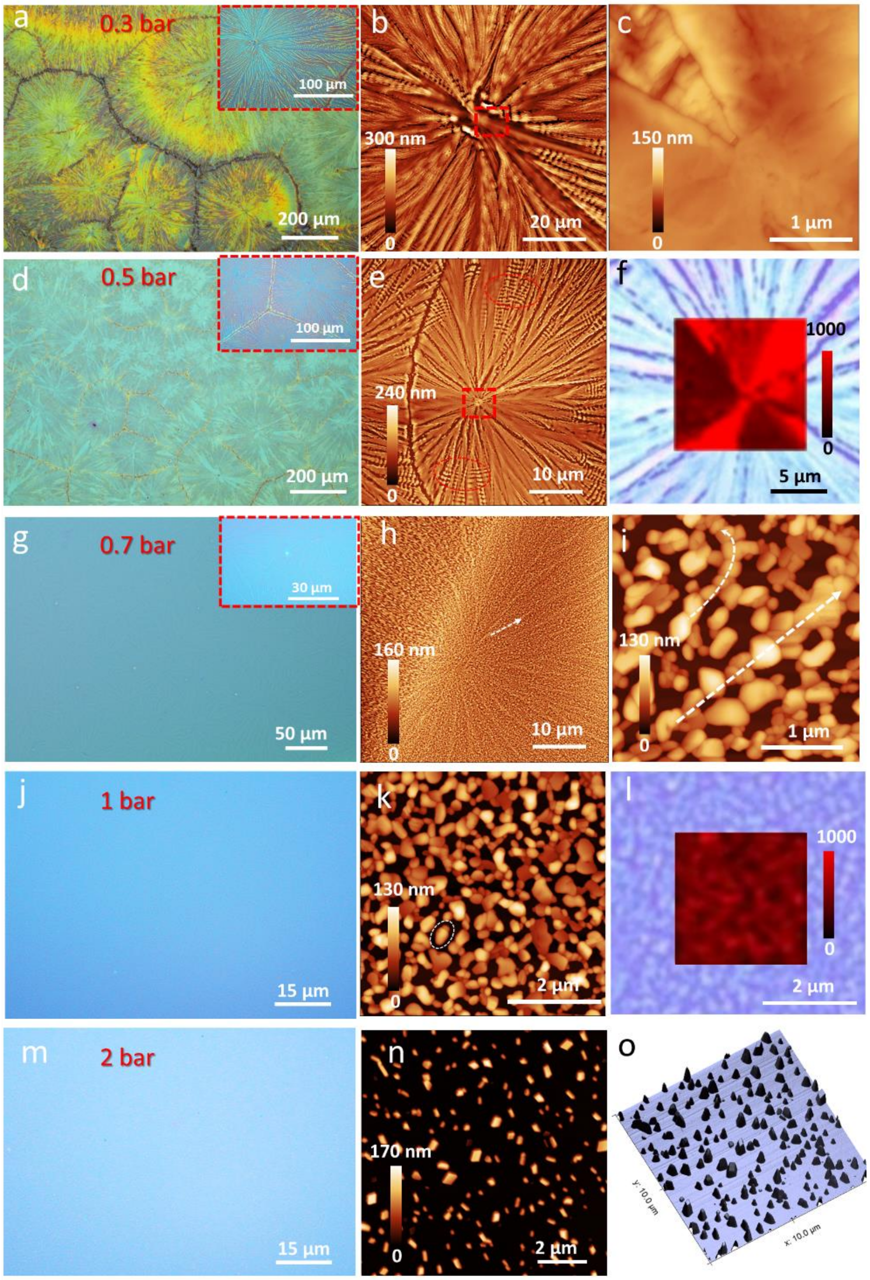

Supplementary Fig. 3. (a) Optical pictures and (b-c) AFM image of glycine crystals deposited by gas blowing coating at pressure of 0.3 bar. The red square in panel b shows the center of the spherical crystal. (d) Optical pictures and (e) AFM and (f) Raman mapping image of glycine crystals deposited at 0.5 bar. (g) Optical pictures and (h-i) AFM image of glycine crystals deposited at 0.7 bar. (j) Optical and (k) AFM and (l) Raman mapping image of glycine crystals deposited at 1 bar. (m) Optical and (n, o) AFM images of glycine crystals deposited at 2 bar.

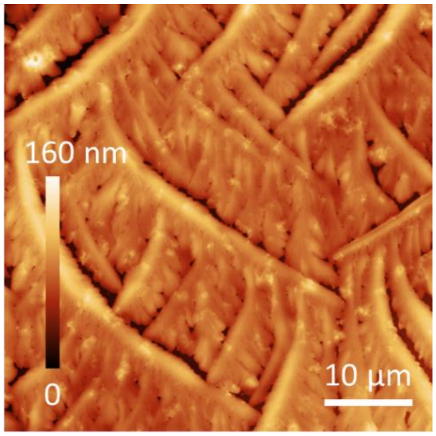

Supplementary Fig. 4. AFM image of glycine crystals obtained by drop casting of 0.01 M glycine aqueous solution on Ar plasma treated SiO_2/Si substrate.

Supplementary Section 2. Characterization of glycine crystals obtained by gas blowing coating

Supplementary Section 2.1. Glycine crystals deposited at different pressures

AFM measurements were performed on an area of 8x8 μm^2 on samples made by gas blowing 0.01 M glycine solutions at pressure of 1 bar (**Supplementary Fig. 5a**) and 1.5 bar (**Supplementary Fig. 5b**). More than 200 crystals were measured: their thickness and lateral size are reported in **Supplementary Table 1**. Raman maps were performed also in the same area: **Supplementary Fig. 5c** shows the intensity of the at 2953 cm^{-1} C-H stretching mode of glycine crystals obtained at 1 bar; **Supplementary Fig. 5d** shows the intensity of the at 2972 cm^{-1} C-H stretching mode of glycine crystals obtained at 1.5 bar. The Raman spectra indicate that the crystals obtained at 1 bar are all β-glycine, while those obtained at 1.5 bar are all α-glycine.

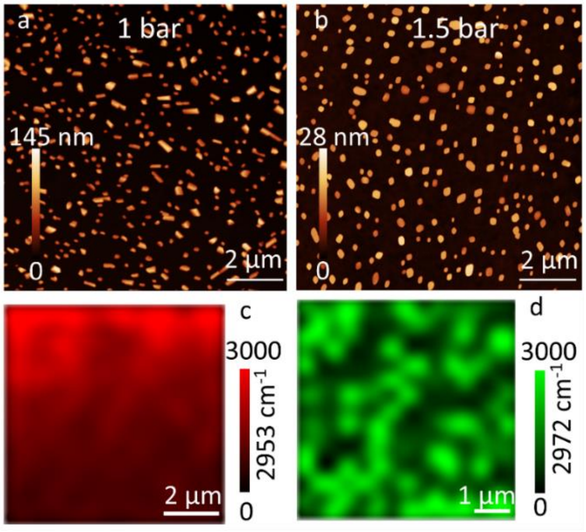

Supplementary Fig. 5. AFM images of the glycine crystals obtained by gas blowing coating of 0.01 M glycine solution at: (a) 1 bar and (b) 1.5 bar. The corresponding Raman maps of the intensity of symmetric C-H stretch Raman mode of glycine crystals obtained at: (c) 1 bar and (d) 1.5 bar.

Supplementary Table 1. Thickness and lateral size derived from the AFM images of the glycine crystals obtained at different pressure extracted from Supplementary Fig. 5**.**

	Number of crystals	Mean	Standard Deviation	Minimum	Median	Maximum
**1 bar**						
Thickness (*h*)/nm	302	56	9	38	54	86
Lateral size (*L*)/nm	302	264	74	131	246	536
Aspect ratio, *L*/*h*	302	4.7	0.8	3	4.5	7
**1.5 bar**						
Thickness (*h*)/nm	253	15	1.7	10	14	21
Lateral size (*L*)/nm	253	296	77	158	288	668
Aspect ratio, *L*/*h*	253	20	5	12	19	46
Flatness ratio, *S*/*M*	253	0.07	0.02	0.03	0.07	0.14
Elongation ratio, *M*/*L*	253	0.72	0.09	0.41	0.72	0.93

Supplementary Section 2.2 Glycine crystals deposited by using different concentrations at 1.5 bar

Supplementary Section 2.2 Glycine crystals deposited by using different concentrations at 1.5 bar

AFM measurements were performed on an area of 20x20 μm^2 on crystals made with starting concentration of 0.1 M (**Supplementary Fig. 6a**) and 5x5 μm^2 for crystals made with starting concentration of 0.0025 M (**Supplementary Fig. 6b**). More than 200 crystals were scanned: their thickness and lateral size are reported in **Supplementary Table 2**. Raman maps were performed also in the same area: **Supplementary Fig. 6d** and **d** show the intensity of the symmetric C-H stretching mode of glycine at 2953 cm^{-1} and 2972 cm^{-1} for the samples produced with 0.1 M and 0.0025 M, respectively. The Raman analysis indicates that the crystals obtained at 0.1 M are β-glycine, while those obtained at 0.0025 M are α-glycine. **Supplementary Fig. 7** shows details of the AFM analysis performed on the two samples: remarkably, the crystals morphology obtained using 0.0025 M are rather different from those obtained using 0.01 M (see main text). A further decrease in glycine concentration, down to 0.0005 M (**Supplementary Fig. 8**), gives rise to glycine dots with the thickness of less than 2 nm and size below 50 nm, randomly isolated, and likely attributed to individual nuclei.

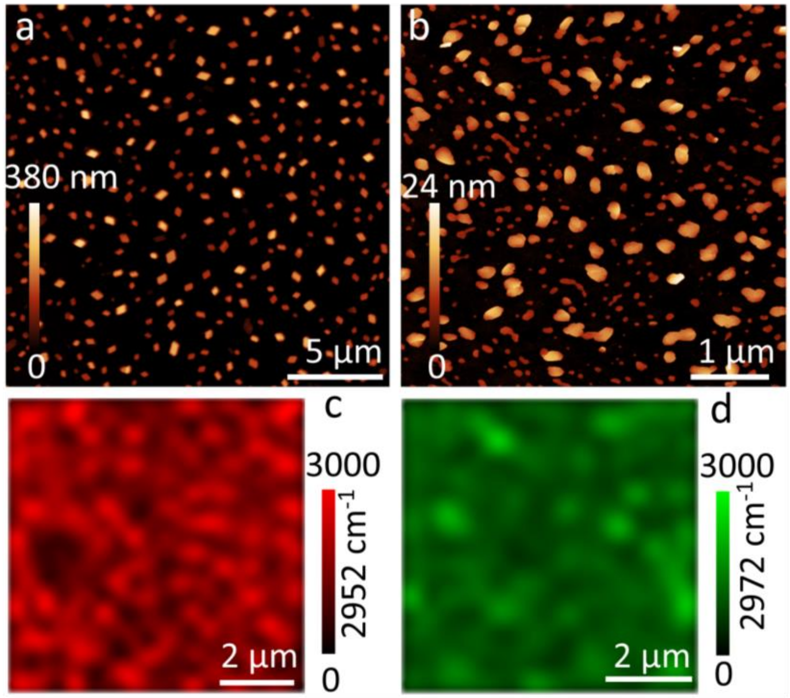

Supplementary Fig. 6. AFM images of the glycine crystals deposited by gas blowing coating using a starting concentration of: (a) 0.1 M and (b) 0.0025 M glycine aqueous solution at a pressure of 1.5 bar. (c) Raman maps of the intensity of the symmetric C-H stretch peak of glycine crystals deposited by gas blowing coating using a starting concentration of 0.1 M. (d) Raman maps of the intensity of the symmetric C-H stretch peak of glycine crystals deposited by gas blowing coating using a starting concentration of 0.0025 M.

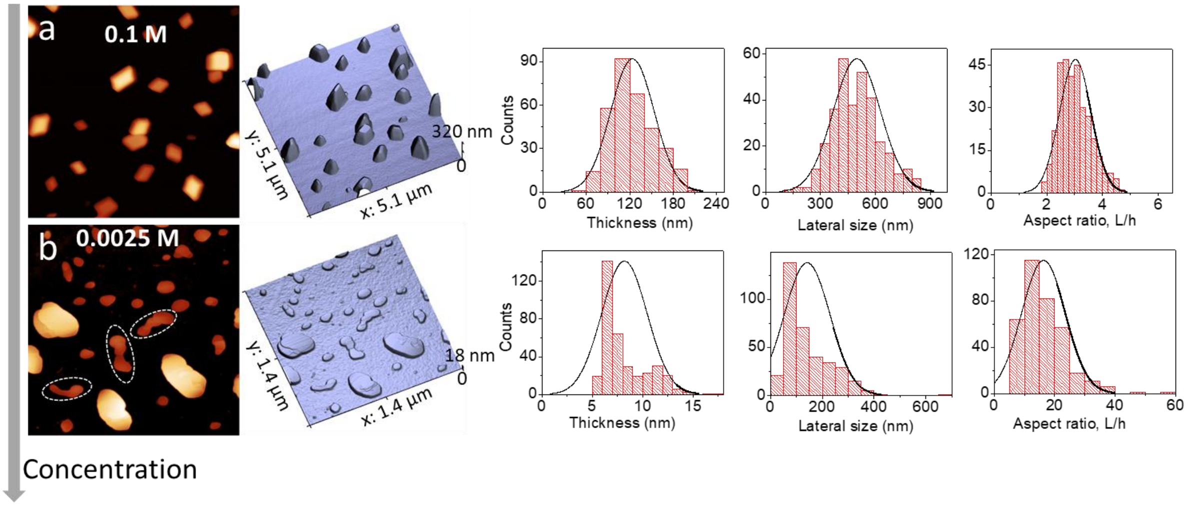

Supplementary Fig. 7. (from the left to the right panels) AFM images, 3D height profile and the statistics of thickness (*h*), lateral size (*L*) and aspect ratio (*L/h*) of the glycine crystals deposited by gas blowing coating of 0.1 M (a) and 0.0025 M (b) glycine aqueous solution at a pressure of 1.5 bar, respectively.

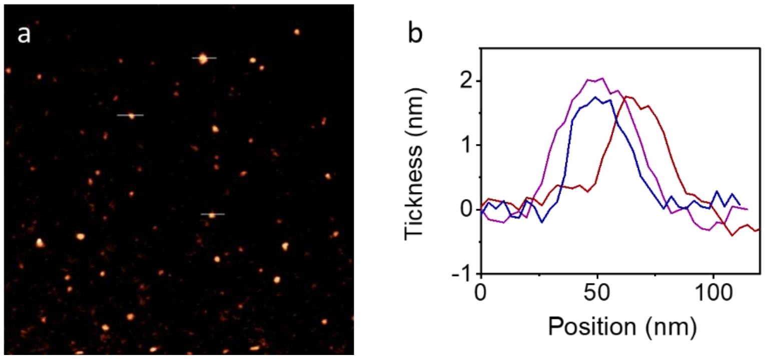

Supplementary Fig. 8. (a) AFM image and (b) height profile the glycine dots indicated in panel a, deposited by gas blowing coating using a starting concentration of 0.0005 M glycine aqueous solution at a pressure of 1.5 bar.

Supplementary Table 2. Thickness and lateral size derived from the AFM images in Supplementary Fig. 6.

	Number of crystals	Mean	Standard Deviation	Minimum	Median	Maximum
**0.1 M**						
Thickness (*h*)/nm	326	124	30	55	119	208
Lateral size (*L*)/nm	326	498	129	123	490	835
Aspect ratio, L/h	326	4	0.6	2.2	4.0	5.6
**0.0025 M**						
Thickness (*h*)/nm	355	8	2	6	7	17
Lateral size (*L*)/nm	355	142	89	41	110	657
Aspect ratio, *L*/*h*	355	16	7	6	15	58

Supplementary Section 2.3. Stability of glycine nanocrystals

Supplementary Fig. 9 shows the Raman spectrum of a β-glycine crystal as-deposited and after 9 months from the synthesis. No appreciable changes are observed.

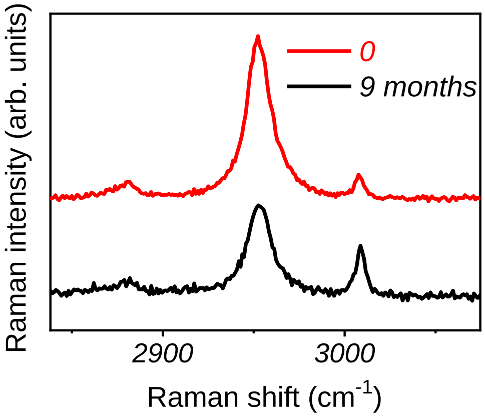

Supplementary Fig. 9. Representative Raman spectra of the as-made glycine crystals and on the same crystals after 9 months of storage. The crystals have been deposited by using a concentration of 0.01 M glycine aqueous solution on SiO_2/Si substrate at a pressure of 1 bar.

Supplementary Section 3. Computer modelling of glycine

Supplementary Section 3.1. Intermolecular energies of glycine polymorphs calculation

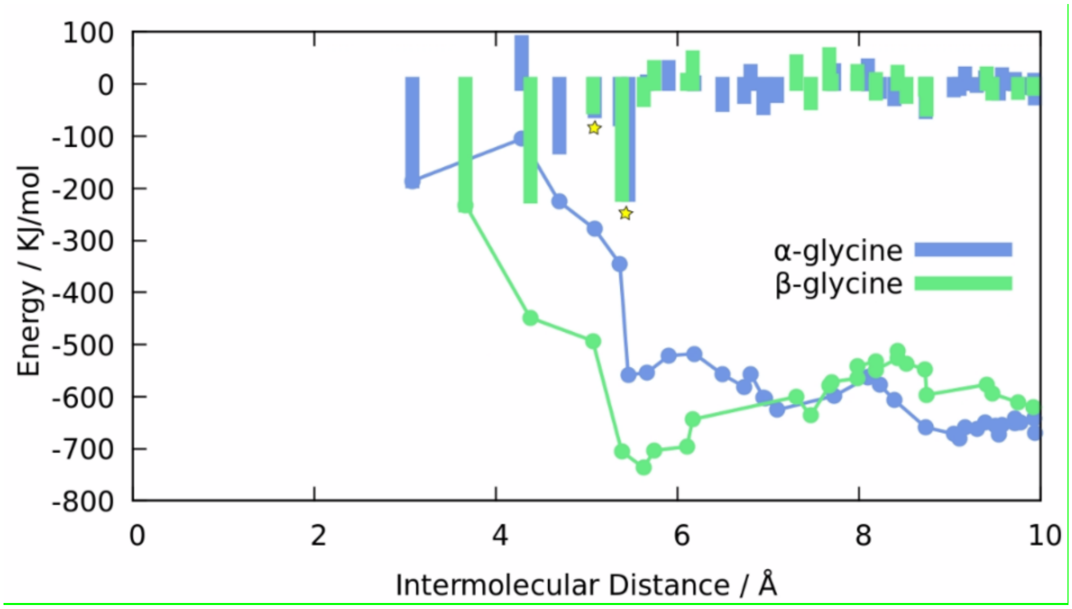

Supplementary Fig. 10. Intermolecular energies (boxes) at different distances with stars marking intralayer contacts and its summation for α-glycine and β-glycine.

Supplementary Section 3.2 α-glycine morphology mapping by CrystalGrower

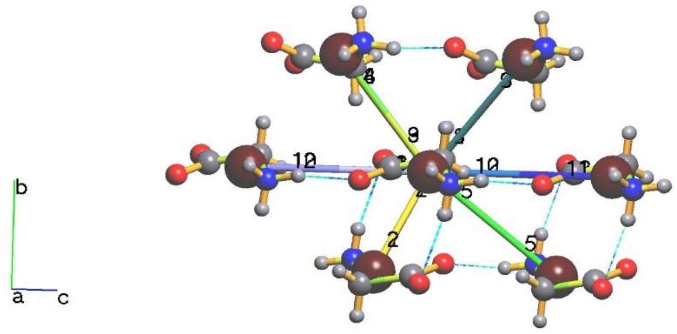

Supplementary Fig. 11. Molecular structure showing the 11 nearest neighbor interactions (NNI) for α-glycine. C, big dark grey spheres, H, small grey spheres, O, red spheres, N, blue spheres.

Supplementary Table 3. Visualisation of the 7 symmetry unique NNIs of α-glycine. This is in order of centroid-to-centroid distance starting from closest to further centroid.

Identity	NNI	Identity	NNI
NNI-A	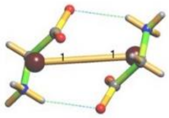	NNI-B	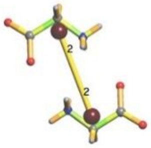
NNI-C	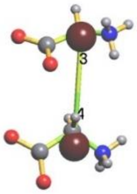	NNI-D	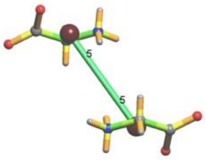
NNI-E	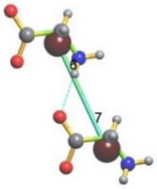	NNI-F	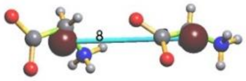
NNI-G	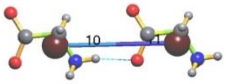		

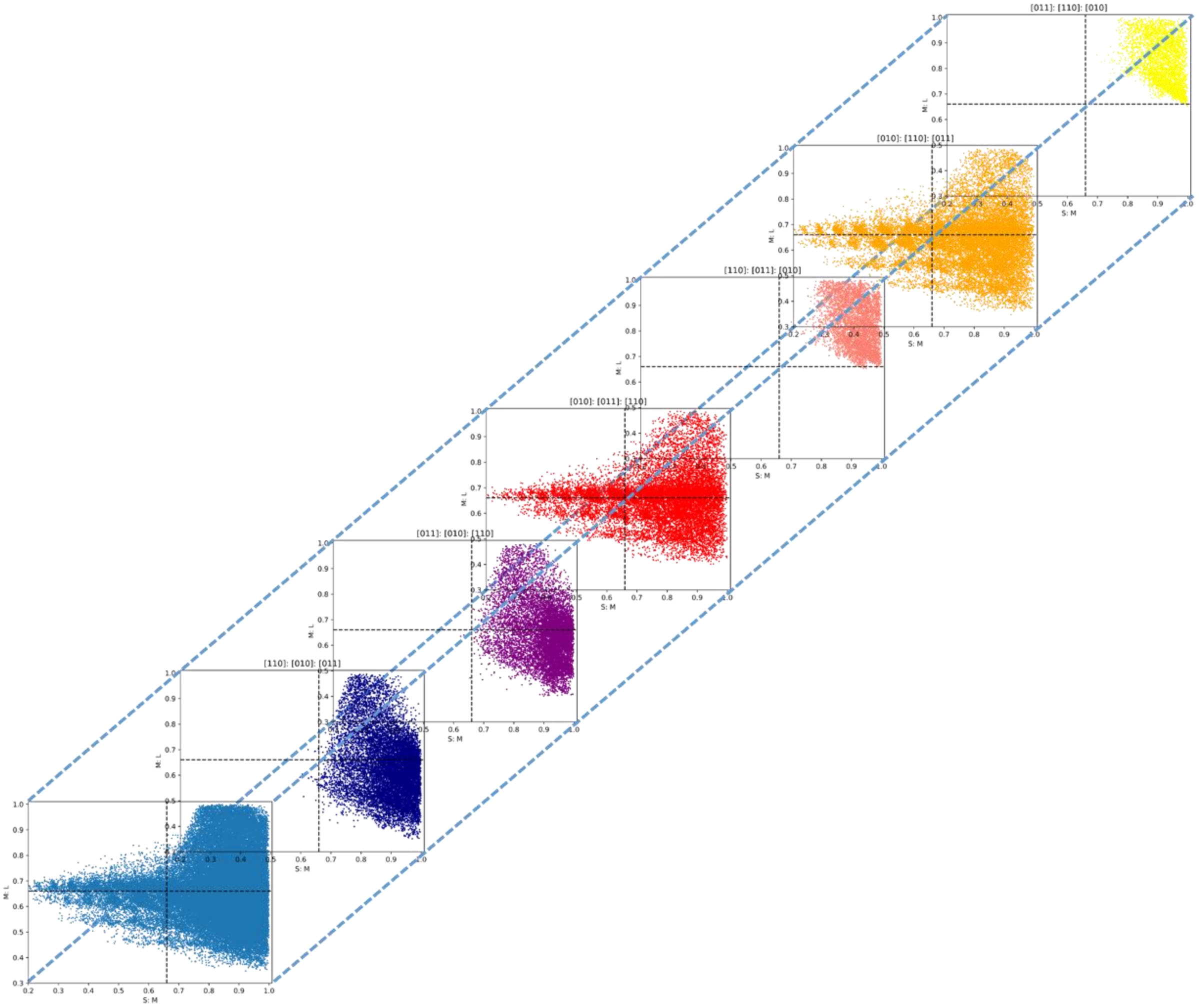

Supplementary Fig. 12. Morphology map of α-glycine crystals filtered by CDA Equations that showing the different growth directions and dominant facets between [110], [010] and [011]. Zingg diagram of all crystals before filtering (the front panel with light blue points), and after filtered by CDA Equations (panels from dark blue to yellow).

Supplementary Table 4. Percentage of all the possible shapes (lath, plate, block and needle) of α-glycine in Zingg diagram filtered by CDA Equations as shown in Supplementary Fig. 12. It can be seen that 99% of all laths or plates that could exist has the [010] direction to be the shortest distances between facets.

CDA Equations S: M: L	Percentage of Shape			
	Lath	Plate	Block	Needle
[110]: [010]: [011]	0.45	0.11	16.29	29.13
[011]: [010]: [110]	0.09	0.00	14.76	21.18
[010]: [011]: [110]	38.8	45.50	19.69	20.37
[110]: [110]: [010]	0.00	0.00	15.68	0.02
[010]: [110]: [011]	60.62	54.40	21.87	29.29
[011]: [110]: [010]	0.00	0.00	11.72	0.01

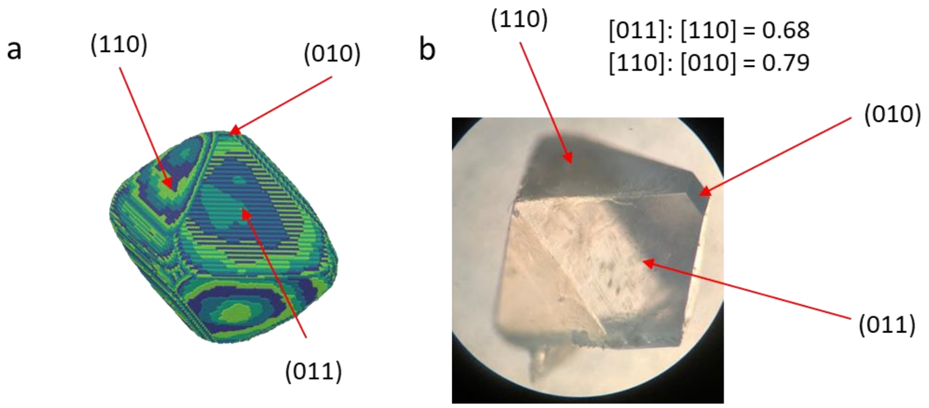

Supplementary Fig. 13. An example of a simulated crystal (a) that matches experimental crystal (b) of α-glycine with [011]:[110] = 0.68 and [110]:(010) = 0.79. This crystal is a block-like crystal with dominant (011) and (110) facets that grown at a high supersaturation of 1.5.

Supplementary Table 5. The corresponding average energies of the NNIs for the same aspect ratio of the experimental crystal in supplementary Fig. 13. The table shows that NNI-D is the most, while NNI-G is the least favorable growth unit in this case.

NNI	ΔG^{NNI}/kcal mol^{-1}	Std. dev.
NNI-A	2.62	0.36
NNI-B	1.82	0.63
NNI-C	1.78	0.66
NNI-D	2.69	0.32
NNI-E	1.47	0.36
NNI-F	2.10	0.63
NNI-G	1.34	0.31

Supplementary Fig. 14 shows the calculated growth and dissolutions rates of α-glycine from water solutions with increasing and decreasing supersaturations. From the growth rate calculations and the calculated morphologies (Supplementary Table 4), it is revealed that the glycine nanosheets would most likely be the result that the (010) facets have been halted because of the confined crystallization from gas blowing.

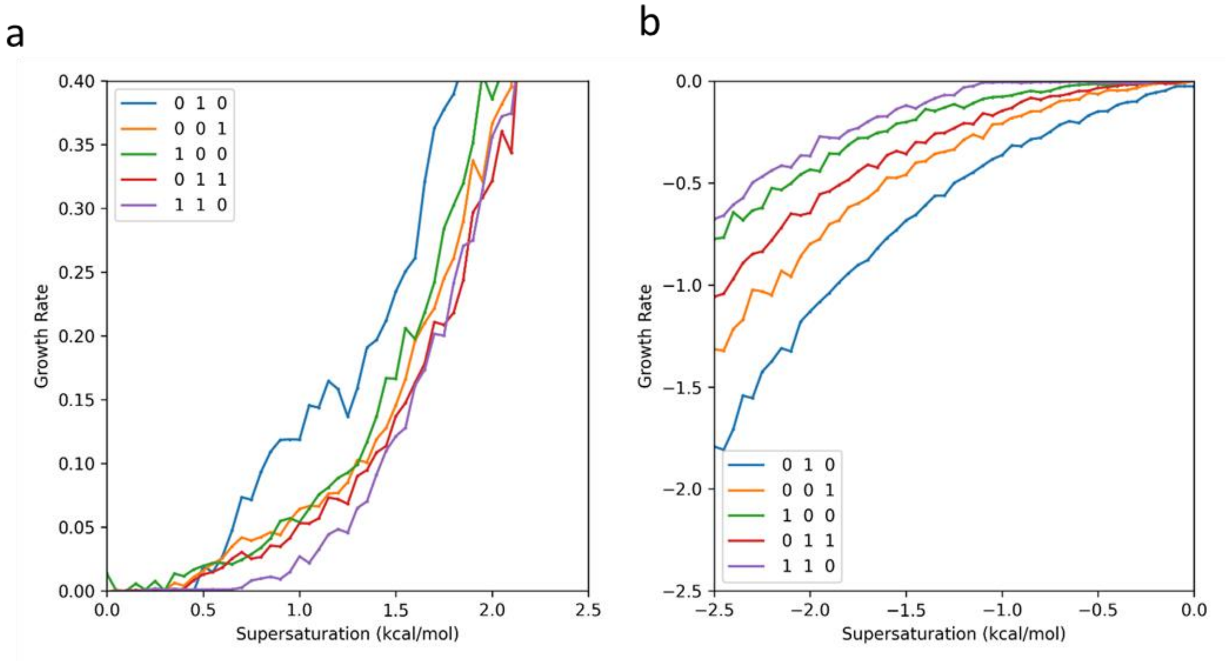

Supplementary Fig. 14. Growth (a) and dissolution (b) rates of α-Glycine crystal grown from water with different directions. Both calculations here assume that the volume is infinite but not finite like which was performed experimentally by gas blowing.

Supplementary Section 4. Gas blowing coating of other organic molecules

Supplementary Section 4.1. Benzamide

Supplementary Fig. 15a shows that drop casting of 0.1 M solution produces large dendritic crystals. **Supplementary Fig. 15b** shows representative Raman spectra of the crystals. Two polymorphs can be identified by the characteristic peaks at 117 cm^{-1}, attributed to form-I benzamide and the peaks at 106, 1110 and 1161 cm^{-1}, attributed to form-II benzamide[2]. Form-I benzamide has similar structure of α-glycine, i.e. hydrogen-bonded bilayers are hold together by VdW interactions, while form-II is a H-bonded 3D structure (**Supplementary Fig. 15c**).

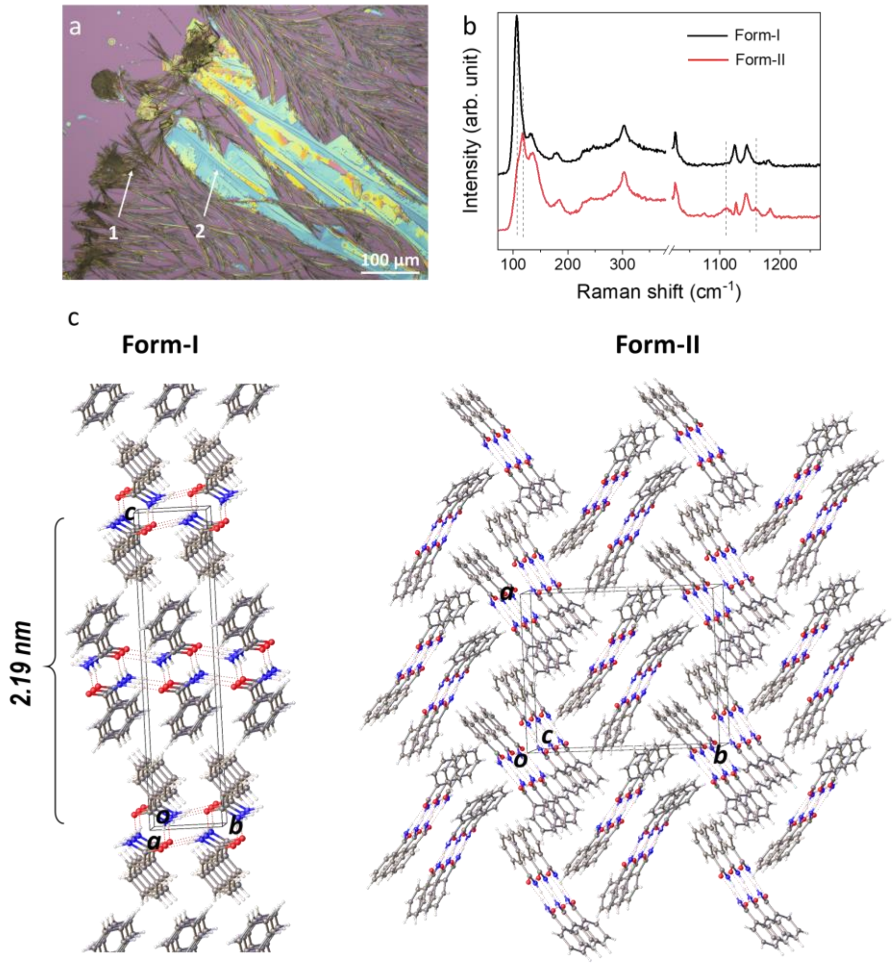

Supplementary Fig. 15. (a) Optical image of benzamide crystals obtained by drop-casting of 0.1 M solution on Ar plasma treated SiO_2/Si substrate. Numbers 1 and 2 indicate two crystals of different polymorphs. (b) The corresponding Raman spectra of crystal 1 (form-II benzamide) and crystal 2 (form-I benzamide). (c) 3D molecular packing of form-I and form-II of benzamide. C: grey, H: white, N: blue, O: red; intermolecular hydrogen bonds are depicted by red dashed lines.

Supplementary Fig. 16a shows representative crystals obtained by gas blowing of 0.01 M benzamide solution onto a SiO_2/Si substrate at a pressure of 1.5 bar. Raman spectroscopy shows that the crystals correspond to form-I benzamide (**Supplementary Fig. 16b**). AFM characterization shows the nanoplate-like morphology of the crystals. Furthermore, a single step terrace of ~1.2 nm (as indicated by the lines in the corresponding AFM cross sections) can be clearly identified (**Supplementary Fig. 16c and d**).

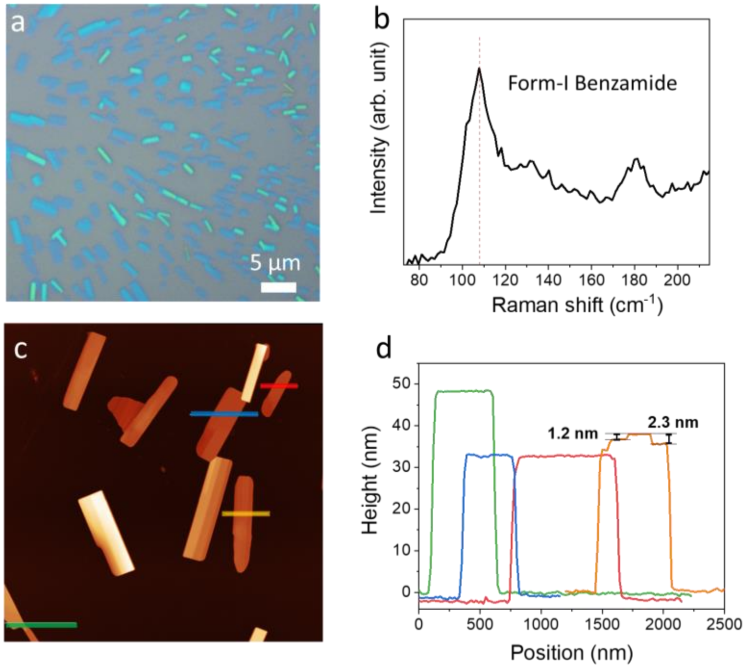

Supplementary Fig. 16. (a) Optical image of benzamide nanocrystals deposited by gas blowing coating of 0.01 M benzamide solution at a pressure of 1.5 bar. (b) Representative Raman spectrum of the crystals. The peaks at 106 cm^{-1} is fingerprint of the form-I. (c) Representative AFM images showing the nanoplate-like morphology of the crystals and (d) the corresponding height profiles of the selected crystals in panel c.

Supplementary Section 4.2. DL-methionine

DL-methionine usually crystallizes in a concomitant mixture of the metastable α-form and stable β-form from a pure aqueous solution at room temperature[3]. The α- and β-forms have similar crystal structures: hydrogen-bonded bilayers hold together by van der Waals interactions, Figure 3a (main text), hence both crystals have nanoplatelets-like morphology[3-5]. **Supplementary Fig. 17a** shows the crystals obtained by drop casting of 0.2 M solution. **Supplementary Fig. 17b** shows representative Raman spectra from 2 selected crystals showing different polymorphs, as shown by the characteristic peaks of α- and β-forms at 350 and 361 cm^{-1}, respectively[4,5]. A clear difference is seen also at the middle region of the Raman spectrum with characteristic peaks at 745, 762, 1010 and 1040 cm^{-1} and in the C-H region of the Raman spectrum with characteristic peaks at 2867 and 2940 cm^{-1} for the α-polymorph.

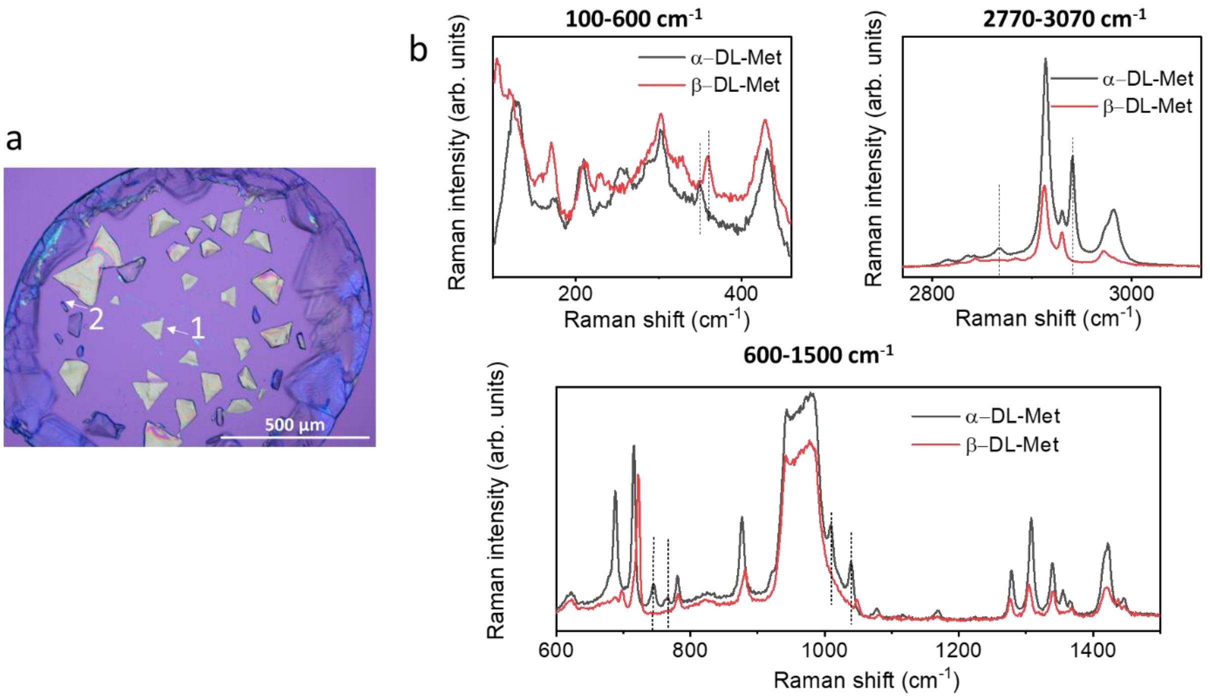

Supplementary Fig. 17. (a) Optical image of DL-Methionine crystals obtained by drop-casting of 0.2 M DL-Methionine on Ar plasma treated SiO_2/Si substrate. Numbers 1 and 2 indicate two crystals of different polymorphs. (b) The corresponding Raman spectra of crystal 1 (α-DL-Methionine) and crystal 2 (β-DL-methionine).

Supplementary Fig. 18a shows representative crystals obtained by gas blowing 0.2 M DL-methionine solution on Ar plasma treated SiO_2/Si substrate at a pressure of 1 bar. AFM characterization shows a clear layered structure, with a single step structure of ~1.7 nm (as indicated by the lines of 1, 2, and 3 in the corresponding AFM cross sections), which corresponds to a bilayer. Raman spectroscopy shows that the nanosheets are α-DL-methionine (**Supplementary Fig. 18b**).

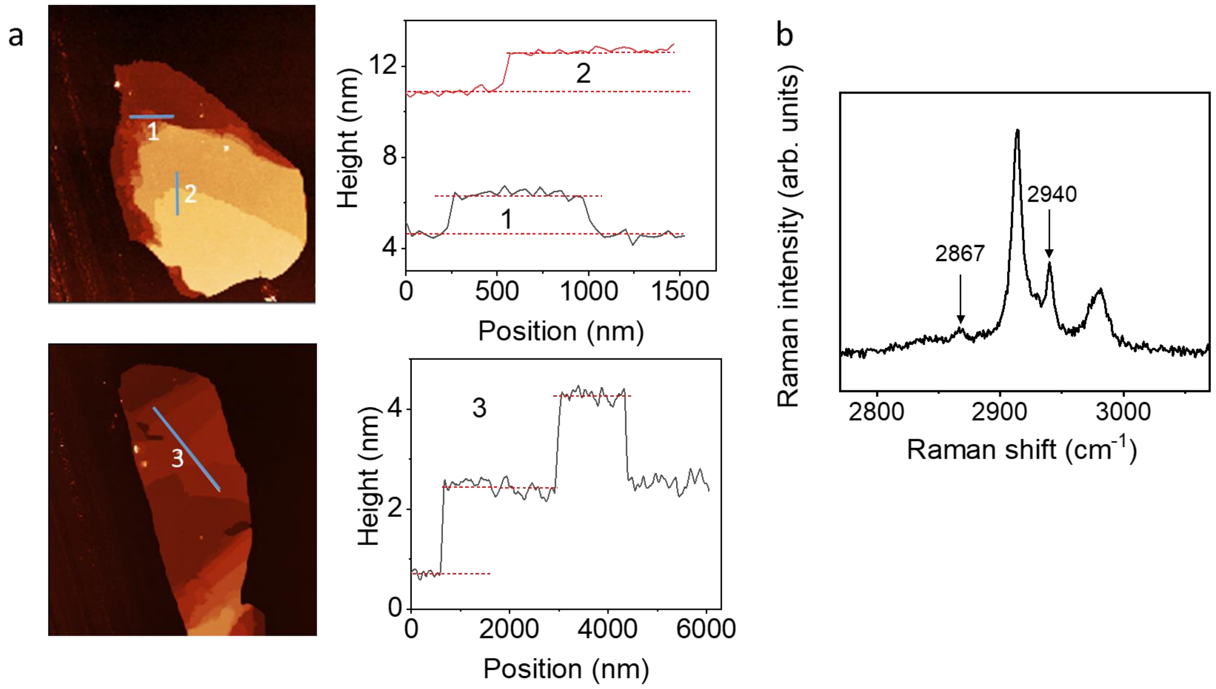

Supplementary Fig. 18. (a) Representative AFM images (left panels) and line cross sections (middle panels) of the DL-methionine crystals deposited by gas blowing of 0.2 M aqueous solution at a pressure of 1 bar. (b) Representative Raman spectrum of the crystals. The peaks at 2867 and 2940 cm^{-1} are fingerprint of the α-form.

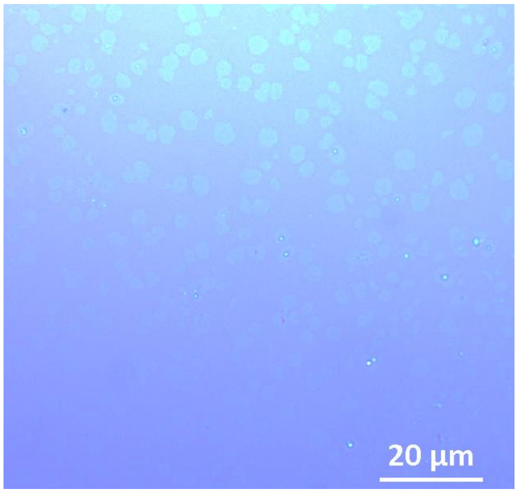

Supplementary Fig. 19. Optical image of DL-Methionine crystals obtained by gas blowing of 0.01 M DL-Methionine at 1 bar on Ar plasma treated SiO_2/Si substrate.

Supplementary Section 4.3. D-mannitol

D-mannitol shows three polymorphs: β, α, and δ, with the phase stability order of β > α > δ under ambient conditions[6]. **Supplementary Fig. 20a** shows the crystals obtained by drop casting. **Supplementary Fig. 20b** shows representative Raman spectra of the crystals: by the characteristic peaks at 1054 and 1233 cm^{-1} are fingerprint of the δ-form and β-form, respectively[7].

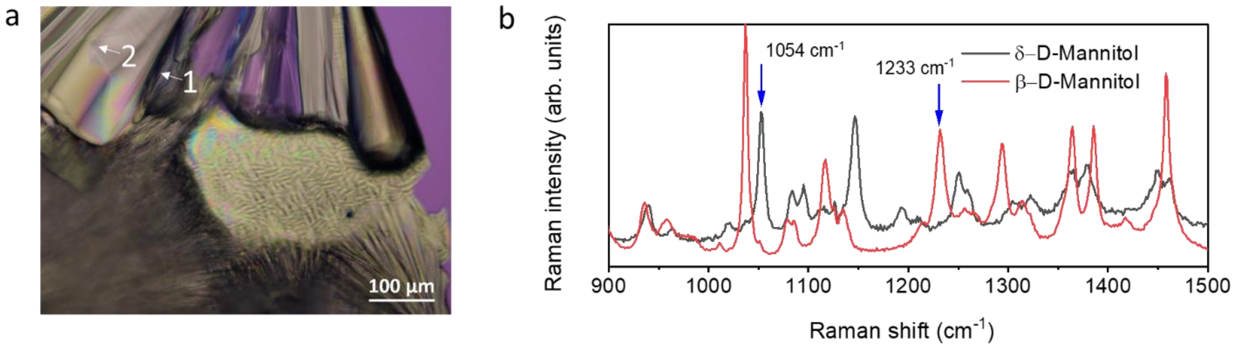

Supplementary Fig. 20. (a) Optical image of D-Mannitol crystals obtained by drop-casting of 0.1 M D-Methionine on Ar plasma treated SiO_2/Si substrate. Numbers 1 and 2 shows two crystals with different polymorph. (b) Raman spectra of crystal 1 and crystal 2. The peak at 1054 cm^{-1} is fingerprint of the δ-D-Mannitol, while the peak centered at 1233 cm^{-1} is fingerprint of β-D-Mannitol.

Supplementary Section 4.4. MOF-5

To prove the universality on producing nanocrystals beyond organic molecules, crystallization onto a surface by gas blowing was applied to a metal-organic framework-5 (MOF-5), which is a prototype material for a series of isoreticular MOFs. **Supplementary Fig. 21a** shows the schematic of the crystal structure, while panels b and c shows the crystals obtained using 0.0006 M precursor solution at the pressure of 1.5 bar onto the SiO_2/Si substrate pre-heated to 100 ° C. AFM confirms the growth of ultrathin crystals with thickness below 30 nm.

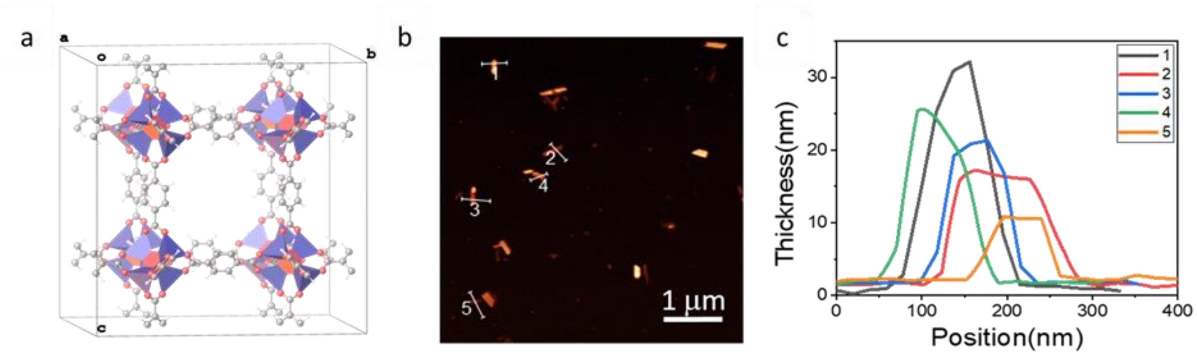

Supplementary Fig. 21. (a) Schematic of MOF-5. On each of the corners is a cluster $[OZn_4(CO_2)_6]$ of an oxygen-centered Zn_4 tetrahedron that is bridged by six carboxylates of an organic linker (Zn, blue polyhedron; O, red spheres; C, black spheres). Hydrogen atoms have been omitted. (b) AFM image of MOF-5 crystals grown by gas blowing of 0.0006 M precursor solution at the pressure of 1.5 bar with the SiO_2/Si substrate pre-heated to 100 °C; (c) Cross section of the crystals 1-5 from panel a, showing the thickness the nanocrystals are below 30 nm.

Supplementary References

1. Tong, J., Doumbia, A., Alieva, A., Turner, M. L. & Casiraghi, C. Gas Blow Coating: A Deposition Technique To Control the Crystal Morphology in Thin Films of Organic Semiconductors. *ACS Omega* **4**, 11657-11662 (2019).

2. Blagden, N. *et al.* Woehler and Liebig revisited: A small molecule reveals its secrets - The crystal structure of the unstable polymorph of benzamide solved after 173 years. *Crystal Growth & Design* **5**, 2218-2224 (2005).

3. Suresh M. & Srinivasan K. Concomitant Polymorphism and Nucleation Control of DL-Methionine Through Antisolvent Crystallization. *Chem. Eng. Technol.* **44**, 614-621 (2021).

4. Shi G. *et al.* Distinct pathways of solid-to-solid phase transitions induced by defects: the case of dl-methionine. *IUCrJ* **8**, 594-594 (2021).

5. Grunenberg A. & Bougeard D. The Observed and Calculated Vibrational-Spectra of Dl-Methionine in the Study of the Solid-State Phase-Transition. *Ber. Bunsen. Phys. Chem.* **90**, 485-492 (1986).

6. Su W. Y., Hao H. X., Barrett M. & Glennon B. The Impact of Operating Parameters on the Polymorphic Transformation of D-Mannitol Characterized in Situ with Raman Spectroscopy, FBRM, and PVM. *Org. Process Res. Dev.* **14**, 1440-1445 (2010).

7. Su W. Y., Hao H. X., Glennon B. & Barrett M. Spontaneous Polymorphic Nucleation of D-Mannitol in Aqueous Solution Monitored with Raman Spectroscopy and FBRM. *Cryst. Growth & Des.* **13**, 5179-5187 (2013).
